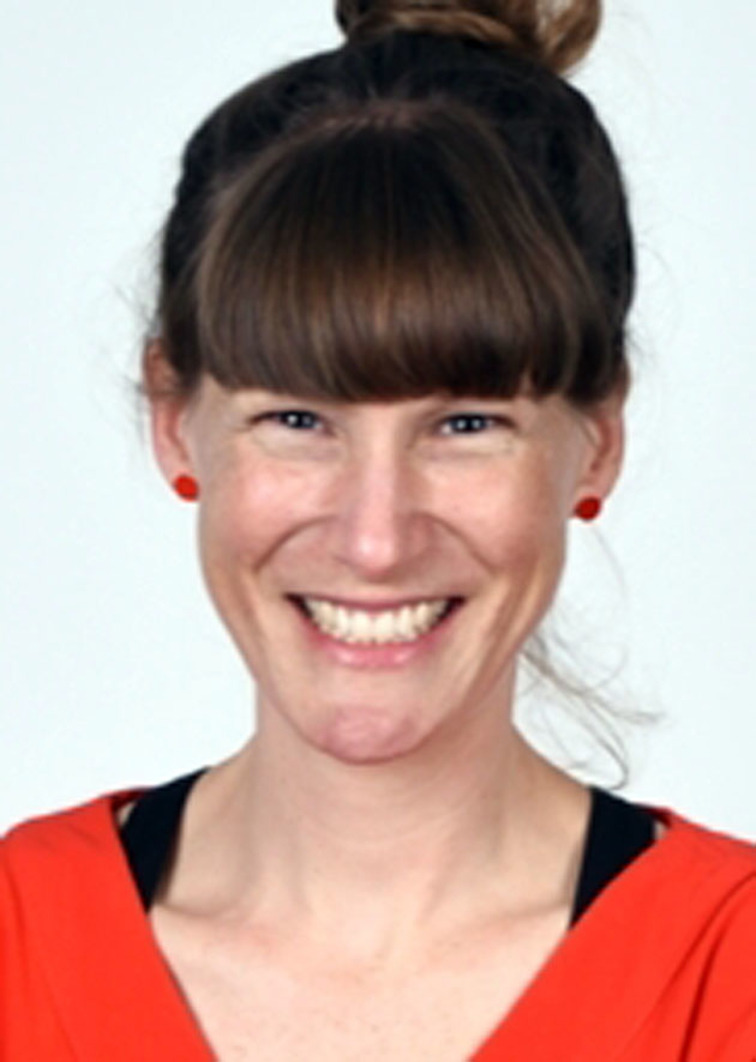# Oligo Pools as an Affordable Source of Synthetic DNA for Cost‐Effective Library Construction in Protein‐ and Metabolic Pathway Engineering

**DOI:** 10.1002/cbic.202100507

**Published:** 2021-12-07

**Authors:** Bastiaan P. Kuiper, Rianne C. Prins, Sonja Billerbeck

**Affiliations:** ^1^ Molecular Microbiology Groningen Biomolecular Sciences and Biotechnology Institute University of Groningen Groningen The Netherlands

**Keywords:** array-based oligonucleotides, gene synthesis, mutagenesis, protein engineering

## Abstract

The construction of custom libraries is critical for rational protein engineering and directed evolution. Array‐synthesized oligo pools of thousands of user‐defined sequences (up to ∼350 bases in length) have emerged as a low‐cost commercially available source of DNA. These pools cost ≤10 % (depending on error rate and length) of other commercial sources of custom DNA, and this significant cost difference can determine whether an enzyme engineering project can be realized on a given research budget. However, while being cheap, oligo pools do suffer from a low concentration of individual oligos and relatively high error rates. Several powerful techniques that specifically make use of oligo pools have been developed and proven valuable or even essential for next‐generation protein and pathway engineering strategies, such as sequence‐function mapping, enzyme minimization, or *de‐novo* design. Here we consolidate the knowledge on these techniques and their applications to facilitate the use of oligo pools within the protein engineering community.

## Graphical abstract

**The construction** of large variant libraries is critical for rational protein engineering, directed evolution, and protein *de novo* design, but is constrained by the relatively high cost of DNA synthesis. Here, we consolidate methods that allow using array‐synthesized oligo pools as DNA input – a cheap but error‐prone source of DNA.

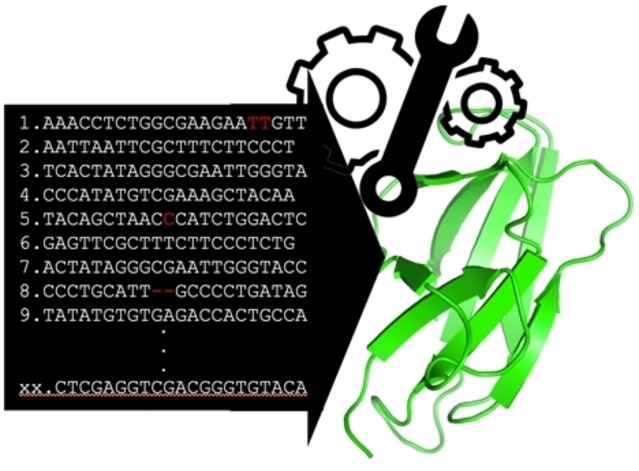

## 1 Introduction

Our capacity to enhance and change the function of proteins or design entirely new ones is essential to unlocking their potential for medicine, green catalysis, and the food and textile industry.

The field of protein engineering has rapidly evolved, and while early techniques are mostly built upon random mutagenesis, currently more targeted approaches are available. The rise of structural data, generation of detailed sequence‐function maps^[1]^ and computational tools,[^2^, ^3^, ^4^, ^5^] combined with next‐generation sequencing technologies,^[6]^ enable scientists to generate smart libraries for directed evolution[^7^, ^8^, ^9^] and to perform rational engineering or to design new proteins from scratch.[^4^, ^5^]

For example, in deep‐mutational scanning (DMS) all residues of a protein are saturation‐mutagenized and then functionally characterized, allowing a protein engineer to create detailed sequence‐function maps of a protein.^[1]^ These fitness maps can identify structurally or functionally important residues that can then infuse further library design. In the context of enzyme engineering, information‐rich sequence‐function maps obtained from such methods allowed researchers to probe the relationship between enzyme fitness and solubility,^[10]^ folding^[11]^ and heterologous expression levels,^[12]^ it has been used to identify sequence determinants of enzymatic substrate specificity,^[13]^ and it has been used to create smart libraries for directed evolution.^[9]^ A collection of available DMS data sets is currently consolidated here: https://www.mavedb.org.^[14]^

Further, computational tools such as artificial intelligence (AI)^[8]^ and *de novo* protein design strategies[^5^, ^15^] currently revolutionize the way we do protein science. For example, AI has been used to guide and accelerate the pace of directed evolution^[2]^ and has recently been used to predict from mostly database‐available sequences which combination of mutations likely yields a functionally optimized protein or enzyme (in respect to a specific user‐set function).^[3]^

While these developments will continue to expand the functionality of proteins in general and enzymes specifically, they also demand the creation of massive variant libraries. For instance, to create a DMS library of a 300 amino acid protein, 5701 gene variants (the wildtype sequence plus 19 amino acid exchanges for all 300 positions) need to be generated. For the functional optimization of a protein or the *de novo* design of a protein – even when using state‐of‐the‐art AI algorithms or the best *de novo* protein design protocols to reduce library size – between ten and a few thousand full genes need to be synthesized and tested to find the desired function.[^3^, ^4^] While massive parallel sequencing of DNA has become very affordable over time, the synthesis of DNA is however still prohibitively expensive when considering the scale needed to build complex libraries.^[16]^ As such, DNA synthesis becomes a new bottleneck for next‐generation protein engineering.

The currently cheapest available source of synthetic DNA is micro‐array‐synthesized oligonucleotides, commercially available as ‘oligo pools’.^[16]^ Oligo pools are mixes of thousands of individually designed polynucleotides of up to 350 bases in length. Traditionally, oligonucleotides have been synthesized by solid‐phase phosphoramidite chemistry.^[17]^ This column‐based synthesis generates up to 200 mers with error rates of 1 in 200, yields of 10 to 100 nmol per product for a cost of 0.05–1 USD per base dependent on the length and concentration yield.[^16^, ^18^, ^19^] These individually synthesized oligonucleotides are then routinely further used for the synthesis of gene‐length DNA fragments using different PCR‐based methods.[^20^, ^21^] To increase throughput and decrease the cost of oligonucleotide synthesis, several technologies have been developed over the last three decades to synthesize oligonucleotides in spatially decoupled microarrays,[^22^, ^23^, ^24^, ^25^] lowering costs by several orders of magnitude (0.00001 to 0.001 USD per base).^[16]^ Microarray‐based technologies allow synthesizing thousands of individual user‐defined sequences, eventually delivered as a pool of molecules.

Even though these microarray‐based oligo pools are cheap, there are several challenges in using them for gene synthesis and library creation.^[16]^ First, while the number of individual user‐defined oligo sequences in a pool is large, their individual concentration is quite low. This challenges their use in traditional gene synthesis protocols, which still mostly rely on oligos from column‐based synthesis. Second, the longer the oligos the higher the percentage of truncated molecules, further lowering the expected concentration of full‐length molecules. Third, the error rates for oligo pools are usually higher than those for column‐synthesized oligos.

Noteworthy, a recently published oligo pool purification method (multiplex oligonucleotide library purification by synthesis and selection (MOPSS))^[26]^ that distinguishes full‐length oligos from oligos with insertions and deletions, will partly overcome this issue if a user is willing to add an extra purification step before library creation.

Early on, oligo pools have been adopted for the creation of guide RNA libraries for functional genomics studies,[^27^, ^28^] for barcoding within screening platforms,^[29]^ and the creation of libraries of short regulatory elements such as promoters,[^30^, ^31^, ^32^] enhancers^[33]^ or silencers.^[34]^ These applications require only short DNA (20 to 100 bases) stretches and can thus manage the shortcomings of oligo pools in limited sequence length, high error rate, and incomplete synthesis.

Libraries for enzyme engineering ideally require the synthesis of error‐free DNA. Each off‐target mutation increases the library size and consequently increases the number of variants that need to be functionally tested to reach full library coverage.

To still leverage oligo pools for library creation – despite their low yield, their short length, and high synthesis errors – several powerful techniques have been developed over the last five years. These techniques managed to use oligo pools for creating DMS libraries,[^35^, ^36^, ^37^] insertion libraries, or for direct in‐lab assembly of gene fragments,[^38^, ^39^] full genes^[39]^ and pathways^[40]^ and, as such, have and will make many next‐generation protein sciences approaches economically feasible for many laboratories.

**Figure 1:**
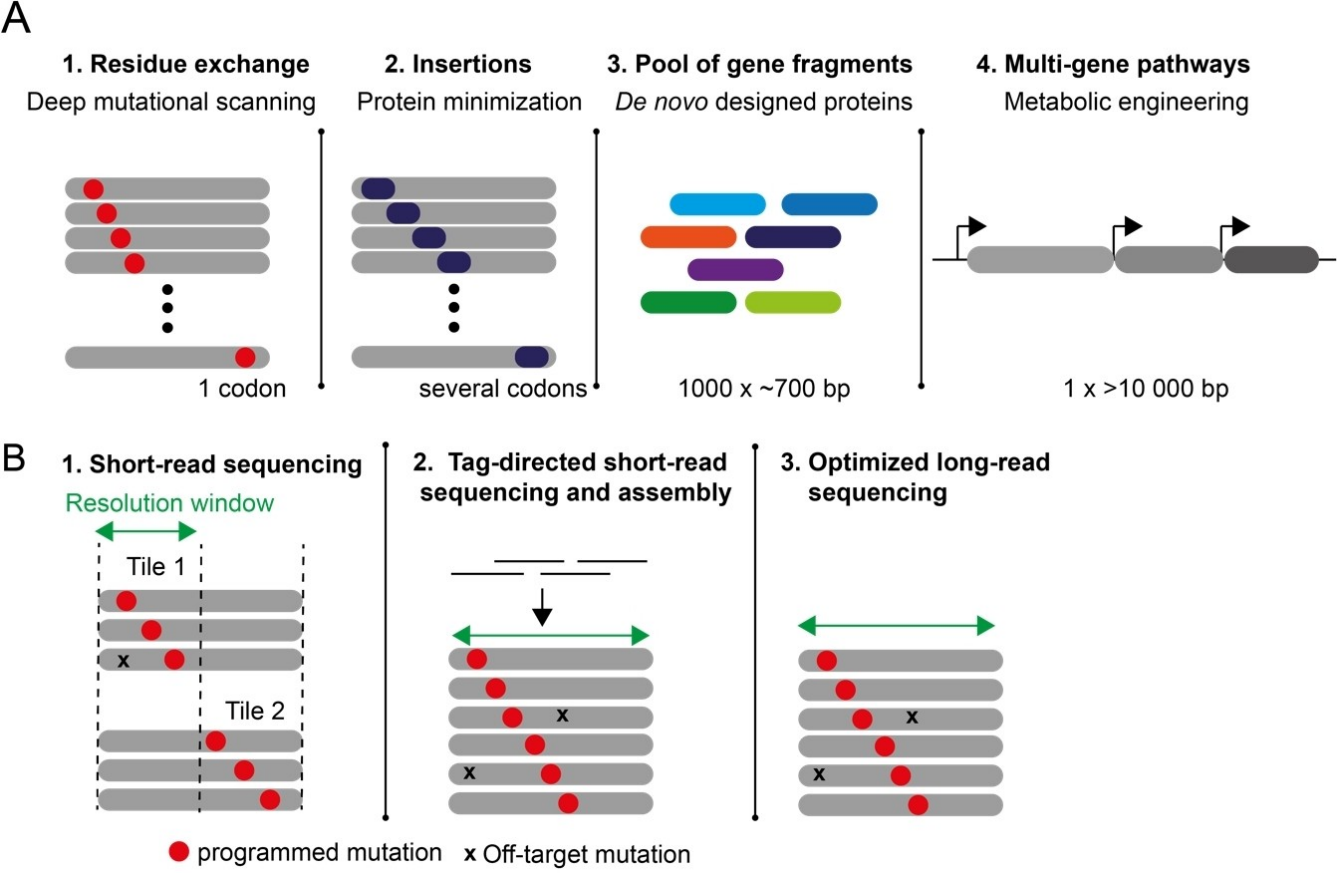
Overview of the discussed methods for mutagenic library creation, gene‐ and pathway assembly, and library analysis. A) Oligo pool‐based methods to generate protein libraries, gene fragment libraries, or pathways. 1) Single residue saturation mutagenic libraries, such as those used for deep‐mutational scanning, can be created from oligo pools by Nicking Mutagenesis (NM),[^35^, ^36^] programmed allelic series (PALS),^[37]^ plasmid recombineering (PR)^[41]^ or CRISPR‐enabled trackable Genome engineering (CREATE).^[42]^ 2) Libraries where short DNA stretches are comprehensively inserted, such as required for protein minimization, have been created by PR and can likely be created by NM, PALS, and CREATE. Single‐residue deletion libraries have been created by PALS. 3) Complex pools of many different proteins fragments can be created by multiple pairwise assembly^[38]^ and DropSynth.[^39^, ^43^] 4) Larger genes and pathways can be assembled as shown by Wan *et al*.^[40]^ B) Library sequence analysis: The quality of libraries needs to be analyzed for the frequency of programmed mutations (red dot) and the frequency of off‐target mutations (cross). 1) Short‐read sequencing – as offered via Illumina‐based services – is highly accurate, high in throughput, and widely accessible but the short read‐length leads to a narrow resolution window. Libraries need to be tiled to be accurately quality controlled, as off‐target mutations outside the resolution window are otherwise invisible.^[6]^ 2) Molecular barcoding and computational assembly can overcome the limited read length in NGS as full‐length sequences of mutagenized clones can be obtained from short NGS reads.^[44]^ 3) Single‐molecule real‐time (SMRT) long‐read sequencing, followed by computational error correction^[45]^ or combined with variant‐concatenation[^46^, ^47^] starts to allow long‐read sequencing for library quality control in protein engineering.

Here we give an overview of these methods to consolidate the knowledge for the protein and enzyme engineering community (Figure 1A).

## 2 Changing Residues: Oligo Pool‐Based Multiple Site‐Saturation Mutagenesis and Deep Mutational Scanning Libraries

Site‐directed mutagenesis has been foundational to protein and enzyme engineering. While commercially available methods like QuikChange™ have long been available to create targeted libraries that mutate single and multiple residues, next‐generation protein engineering technologies require massive parallel changes to a protein sequence. Towards this end, a handful of scalable mutagenesis methods that achieve (near) comprehensive mutagenesis of open reading frames have been developed: PFunkel,^[48]^ Nicking Mutagenesis (NM)[^35^, ^36^] and programmed allelic series (PALS)^[37]^ are *in vitro* methods and plasmid recombineering (PR)^[41]^ and CRISPR‐enabled trackable Genome engineering (CREATE)^[42]^ are *in vivo* methods for use in *E. coli* and the yeast *S. cerevisiae*.

In this review, we highlight those methods (NM, PALS, PR and CREATE), that have been tested to be compatible with the use of array‐synthesized oligo pools rather than individually column‐synthesized and hand‐mixed oligonucleotide pools. Thus, these methods allow for the cost‐effective creation of custom libraries. Noteworthy, even though not explicitly tested in a published format, likely, PFunkel^[48]^ would also work with array‐synthesized oligo pools as it is the precursor technique for the development of NM.

### 2.1 *In vitro* techniques for saturation mutagenesis

Nicking mutagenesis (NM) allows for the one‐pot generation of targeted or comprehensive single‐site or multi‐site saturation mutagenesis libraries in a single day, using regular molecular biology techniques: It requires routinely prepped plasmid DNA, a pool of oligonucleotides, two nicking restriction endonucleases (Nt.BbvCI and Nb.BbvCI), an exonuclease (Exo III), a high‐fidelity DNA polymerase (Phusion) and a ligase (Taq). The only non‐routine requirement is that the plasmid DNA needs to encode the 7 bp BbvCl recognition site, which can be introduced into any plasmid by regular cloning techniques.

The workflow of NM starts by selectively degrading one plasmid DNA strand via the nicking restriction endonuclease Nt.BbvCI and exonuclease treatment. The obtained circular single‐stranded DNA subsequently serves as the template for primers that encode for the desired mutations. The ratio of primer to template allows tuning the number of mutations per gene. If the number of primer molecules is much lower than the number of template molecules, then effectively only one primer can bind per template. Each primer is then extended by a high‐fidelity polymerase and the created strand is ligated with Taq DNA ligase resulting in covalently closed circular double‐stranded DNA. One strand contains the mutations, the other the wild‐type sequence. Subsequent treatment with a second nicking restriction endonuclease (Nb.BbvCI, binds and nicks the reverse complementary Nt.BbvCI recognition site) and exonuclease leads to digestion of the still‐wild‐type strand of the plasmid DNA, yielding a single‐stranded mutated plasmid. Amplification with a universal primer that binds outside the target area for mutagenesis eventually creates a double‐stranded plasmid with the manifested mutations in both strands.

In the original paper describing NM,^[35]^ the authors used a hand‐made mix of column‐synthesized oligonucleotides to establish the method. Later, it became clear that very little primer was needed for the introduction of point mutations and that primers encoded as micro‐array synthesized oligo pools could be used.^[36]^ The authors test the oligo‐pool based NM with three short protein segments of <100 residues (short enough to enable full sequencing via paired‐end reads, see section 5) and show that dependent on the oligo‐concentration, 97.4 to 100 % mutational coverage could be reached after a single round of NM (Table 1). Noteworthy, all 7118 oligos required for mutagenizing all three proteins were ordered together as one single pool. They further show that the use of oligo pools leads to a more even representation of each amino acid when compared to using NNN codon randomized and hand‐mixed column‐synthesized primers.

**Table 1:** Performance overview of single‐site saturation mutagenesis methods (e. g. used for deep mutational scanning).

Method	Protein (# of mutated codons)	% Coverage^[a]^	% 1 NSM^[b]^	% 0 NSM (wild‐type)^[c]^	% >1 NSM (off‐target)^[d]^	Ref.
*in vitro*						
Nicking mutagenesis (NM)	*E. coli* AmiE (70)^[e]^	100	36.4	52.7	10.8	[36]
	*A. thaliana* PYR1 (86)^[e]^	100	26.5	59.4	13.6	[36]
	Anti‐influenza human antibody variable heavy gene UCA9 (99)^[e]^	97.4	14.1	71.3	15.0	[36]
	Phage ΦX174 F capsid protein (421)	100	41.8	28.6	29.5	[50]
	Phage ΦX174 G spike protein (172)	99.9	49.3	29.3	21.4	[50]
PALS	*S. cerevisiae* Gal‐DBD (64)	99.9	47	24	21	[37]
	Human p53 (393)	93.4	33	30	35	[37]

*in vivo*						
Plasmid recombineering (PR)^[f]^	*A. thaliana*‐derived iLOV (110)	99.8	28.8	60.7	10.5	[41]
CREATE (genomic mutagenesis)	*E. coli* GalK (1)	100	56.8^[g]^	22.4^[g]^	n/a	[42]
	*S. cerevisiae* ADE2 (2)	100	95	5	n/a	[42]
	*E. coli* lysine metabolism, 19 genes, 16,300 designed edits^[h]^	22.7 to 61.6	n/a	n/a	n/a	[54]

n/a: not available; [a] Number of actually observed mutations per 100 designed mutations. Note: differences in sequencing depth used for quality control in the different studies influences the number of observed mutations, thus the apparent coverage. [b] Percent of mutants that carry exactly one desired non‐synonymous mutation (NSM). [c] Percent of mutants that do not carry any NSM, thus being wild‐type (non‐edited) variants. Note that CREATE is the only method that counter‐selects for wild‐type via CRISPR‐Cas9 mediated double‐strand breaks. [d] Percent of mutants that carry more than one NSM, e. g. off‐target mutations. [e] The average of two reported independent runs of NM is given (Table 1 in Ref. [36]). [f] Here, hand‐mixed pools of column‐synthesized oligonucleotides were used. It was shown later that PR also works with array synthesized oligo pools.^[53]^ [g] Calculated based on 80 % of clones being edited (based on colorimetric screen) and 71 % of those 80 % being correctly edited (based on sequencing, 0.8×0.71); and 20 % of clones being wild‐type (based on colorimetric screen) plus 3 % of the 80 % phenotypically edited clones (0.8×0.03) being still unedited at the programmed locus. [h] CREATE was developed and applied for multiplexed pathway mutagenesis. The percent coverage refers to the observed coverage range of five test loci that were analyzed in‐depth (Table 1 in Ref. [54]).

Full sequencing analysis of the three test libraries showed that they consisted of 14–37 % plasmids with the designed one non‐synonymous mutation, 52–71 % plasmids with wild‐type sequence (likely due to template molecules that did not participate in or complete the mutagenesis cycle), and 11–15 % plasmids with more than one non‐synonymous mutation (Table 1). The authors observed that a higher melting temperature of a given primer correlated with higher mutational frequency, giving room for optimization of the protocol. The authors further tested if NM could be used for subsequent multi‐site mutagenesis by subjecting the single‐site saturation library to a second cycle of NM. After this second cycle performed with one of the test proteins, the authors show coverage of 79 % of all possible positions. Double mutations were depleted in near adjacent positions, likely because a second oligonucleotide would either not anneal properly or overwrite the mutation from the first oligonucleotide. An optimized protocol for performing multi‐site NM has recently been developed.^[49]^ This protocol uses model‐driven oligo design to achieve >99 % coverage of a multi‐site mutagenized 32.768 membered antibody library (Table 2). The authors further provide an automated workflow for oligo pool design using a protein‐coding sequence as the sole input.

**Table 2:** Performance overview of double‐ and multi‐site saturation mutagenesis methods (e. g. for targeted protein engineering).

Method	Library size	% Coverage	% 1 NSM	% 0 NSM (wild type)	% 2 NSM	% >2 NSM	Ref.
Double‐site mutagenesis							
Nicking mutagenesis (NM)	n/a	79.2	n/a	60.0	n/a	n/a	[36]
Plasmid recombineering	5940	98.0	26.3	32.6	24.5	16.4	[41]

Multi‐site mutagenesis							
Optimized multi‐site nicking mutagenesis (NM)^[a]^	16 384	99.9	n/a	2.58	n/a	n/a	[49]
	32 768	99.4	n/a	0.25	n/a	n/a	[49]

n/a: not available; [a] The average of the two reported independent runs of optimized multi‐site NM is given (Table 1 in Ref. [49]).

Scalability of the single‐site NM method^[36]^ was later shown by saturation mutagenizing the bacteriophage ΦX174,^[50]^ specifically its two main proteins the F capsid protein (421 amino acids scanned) and G spike protein (172 amino acids scanned). The libraries possessed greater than 99 % of all 11.860 programmed mutations (Table 1). All required oligos could be ordered in one pool. To enable the replication of the plasmids in *E. coli*, the genome was divided into 14 non‐toxic and non‐replicative fragments. The F capsid protein and G spike protein were each tiled into two fragments. Golden Gate cloning was then used to assemble the complete ΦX174 mutant genome and generate libraries of infective viruses that could be used in the future to study viral evolution and to engineer bacteriophages for therapeutic applications.

A second method that achieves saturation mutagenesis in one volume is PALS (programmed allelic series).^[37]^ Similar to NM, PALS allows for the generation of targeted or (near)‐comprehensive single‐site or multi‐site saturation mutagenesis libraries using regular molecular biology techniques and no special equipment. It has a few more steps than NM, takes rather two instead of one day to prepare the library and it leads overall to slightly lower coverage than NM (as such, libraries are called near‐comprehensive, reported 98 % coverage after a single round, Table 1). Instead of amplifying the full plasmid that encodes the target region for mutagenesis as done in NM, PALS only uses a PCR amplified target fragment as input. Therefore, PALS might become the method of choice in case the used plasmids are large and full amplification becomes inconvenient (e. g. as usually the case for mammalian vectors) or in case the mutagenic library is supposed to be encoded in the genome of an organism. In that case, the PALS PCR library can directly be used as a homology‐directed repair fragment for genome engineering.

PALS is based on the annealing and extension of mutagenic oligos along a deoxyuracil (dU)‐ marked wild‐type sense strand. The dU‐marking allows the experimenter to specifically digest the wild‐type sense strand after the extension is completed using uracil‐DNA‐glycosylase and exonuclease VIII (also known as USER enzyme mix). Each mutagenic oligo encodes for a unique 3’‐priming site and a common 5’‐priming site which are subsequently used to amplify the extended oligos by PCR creating a single‐mutagenized double‐stranded library (the unique priming sites get cleaved). At this point each library member has a different 3’end, depending on where the mutagenic oligo annealed. To create the full template length, the 5’–3’ strand of the mutagenized amplicons are used as mega primers to extend their 3’ ends along a dU‐marked wild‐type anti‐sense strand.

The required dU‐marked (single‐stranded) sense and anti‐sense strands can be created via PCR using dUs instead of dTs as nucleotides and by using a phosphorylated reverse or forward primer. The phosphorylation of the 5’end is harnessed to digest the antisense or sense strand via lambda exonuclease, leaving a dU‐marked (single‐stranded) sense and antisense strand.

After one round of PALS on two test proteins (the DNA‐binding domain of the yeast transcription factor Gal4, and the full human transcription factor p53) the authors report 93–98 % coverage, with 33–47 % of the molecules showing the correct single mutations, 24–30 % of the molecules being wild type and 21–35 % of molecules having additional mutations on top of the programmed single mutation (Table 1).

### 2.2 *In vivo* techniques for saturation mutagenesis

Here we highlight two techniques, PR^[41]^ and CREATE,^[42]^ that achieve near‐comprehensive saturation mutagenesis directly in the living microbial host *E. coli* (PR) and both, *E. coli* and *S. cerevisiae* (CREATE). *In vivo* techniques are useful, as the libraries can be directly functionally screened without the need for a transformation step.

PR relies on a method called “recombineering”, which, historically has been widely used for genomic engineering in *E. coli*,^[51]^ for example in a process called multiplexed automated genome engineering (MAGE) that mutagenized the genome via oligo recombineering for functional genomics and metabolic engineering.^[52]^ Higgins and coworkers demonstrated that recombineering can also be used to mutagenize regions of plasmids, e. g. regions encoding for proteins.

PR is based on incorporating synthetic oligonucleotides directly into a gene of interest via the help of lambda phage protein ß‐mediated recombination.[^51^, ^52^] Mechanistically this (likely) involves that the ß protein binds to an oligonucleotide and directs it to the lagging strand at the replication fork of replicating DNA. The oligo is subsequently incorporated into the growing strand, thus allowing the experimenter to edit the new DNA molecule in a programmable fashion.

The procedure is cheap, as it does not involve enzymes or specialized equipment, and is simple, just involving a transformation and overnight growth selection for plasmid transformants: In brief, electro‐competent *E. coli* cells are co‐transformed with the plasmid of interest and a pool of oligos with user‐defined sequences (60 bp, targeting the lagging strand and encoding the mutagenic codon in its middle). After the electroporation cells are recovered for several hours without antibiotic selection, followed by overnight growth under plasmid‐selective conditions. Plasmids can then be miniprepped to recover the library or one can directly continue with functional selection for the desired protein phenotype. Higgins *et al*. show that a nearly comprehensive mutational‐scanning library can be achieved in a single transformation. They found 99.8 % of all possible single amino acid conversions represented, for their 110‐residue model protein iLOV (Table 1). Similar to the *in vitro* methods, no selection against wild‐type plasmid is included in the method and they find that about 29 % of their plasmids have a single mutation while over 61 % of all plasmids are the wild type (Table 1). Five subsequent rounds of PR lowered the frequency of finding wild‐type plasmids to 26 %, while 33 % of colonies had a single mutation and 41 % of plasmids had two or more mutations. As such, the authors also show that multiple rounds of PR (five rounds in the reported case) can be used to create targeted double‐site saturation libraries with high coverage (98 %, Table 2).

In their first proof‐of‐concept of PR, Higgins *et al*. use a mix of 110 column‐synthesized oligonucleotides rather than array‐synthesized oligo pools, but in a later study, they show that amplified oligo pools can be used for PR mutagenesis.^[53]^

As a technical remark, PR should theoretically work with any template that replicates in *E. coli*, but it needs to be performed in a suitable strain background that encodes and expresses the Lambda Red system (including the ß protein) and carries a deletion for MutS. The MutS deletion is required to inactivate the mismatch repair system in *E. coli* and allow for effective recombination.^[52]^ The *E. coli* strain EcNR2 which is widely used for recombineering is available via Addgene (#26931).^[52]^

A second method that uses array‐synthesized oligo pools as a starting point for *in vivo* saturation mutagenesis is CREATE.^[42]^ In comparison to PR, which acts upon plasmid‐encoded targets, CREATE is designed to act upon chromosomally encoded proteins and full pathways and allows for pathway‐ or even genome‐wide deep mutational analysis. Mutagenesis is based on inducing a genomic double‐strand break in a protein of interest via CRISPR/Cas9 and allowing the cell to repair the break via homologous recombination with a short homology arm (up to 120 bp) that encodes the programmed mutation and a synonymous mutation in the protospacer adjacent motif (PAM) to prevent future cleavage. Both, the guide RNA (gRNA), which programs the cut site, and the homology arm are encoded together on a plasmid as a so‐called CREATE cassette. The CREATE cassette library (library of all gRNAs and repair arms, typically 10^4^ to 10^6^ members) is thereby designed computationally and ordered as an array‐synthesized oligo pool, amplified by PCR, and cloned into a cassette vector. The covalent linkage of the gRNA with the programmed edit on the homology arm allows the experimenter to use the CREATE cassette as a plasmid‐encoded barcode during future selections, assuming that frequency of changes in a CREATE plasmid and the edited genome frequency stay coupled during growth.

The authors first test CREATE by inactivating the *galK* gene of *E. coli* by introducing a single nonsense mutation. Depending on the length of the used homology arm (80 to 120 bp) and the distance of the mutagenized site from the PAM (17 to 59 bp), CREATE achieves editing efficiencies of 75 to 90 %, as judged by a *galK*‐based colorimetric assay. Sequencing revealed that 71 % of those edited clones carried the designed nonsense mutation plus the designed synonymous mutation in the PAM sequence, 26 % contained only the PAM edit, and 3 % showed neither of the designed edits at this locus (Table 1).

Further, CREATE could be functionally transferred into the yeast *S. cerevisiae*. Editing of the *S. cerevisiae ade2* gene to introduce a tandem stop codon showed editing efficiencies of 95 % based on a colorimetric read‐out, with 100 % of those phenotypic hits being correctly programmed (Table 1).

In a follow‐up study, the authors show the scalability of CREATE by deep mutagenesis of an entire metabolic network.^[54]^ Specifically, the authors designed 16,300 mutations within the binding‐pockets of 19 enzymes or transporters, that comprise four primary routes that guide lysine flux in *E. coli*. Using NGS analysis of four test loci, the authors show the library coverage to range between 22.7 to 61.6 %, indicating high editing efficiencies given the size of the library (Table 1). Eventually, by mutationally perturbing *E. coli*’s lysin metabolic network and subsequently challenging this library with the antimetabolite AEC, the authors could evaluate in parallel the contribution of these 16,300 targeted mutations toward antimetabolite resistance and thus overall pathway flux. As such, the authors could (near) comprehensively map sequence‐function relations that alter the pathway's function, setting a framework for investigating complex multigenic phenotypes.

### 2.3 Further considerations for saturation mutagenesis

In summary, NM, PALS, PR and CREATE are effective ways to create (near‐)comprehensive single‐site or multi‐site mutagenic libraries with the use of oligo pools. For library screening and associated library coverage calculations, it needs to be kept in mind that all methods lead to libraries that show only ∼50 % or less of the total molecules to be desired single mutations, and contain a significant amount of wild‐type plasmid or plasmids with non‐desired mutations (Table 1 and 2). Mutation efficiency might not matter in case a high‐throughput selection is available, but might be limiting for protein engineering efforts that require expensive or laborious screens as the number of screened mutants needs to be doubled to cover such a library (compared to a 100 % mutagenized library).

## 3 Encoding Short DNA Stretches: Oligo Pool‐Based Insertion and Deletion Scanning Libraries

Besides changing single amino acids, several protein engineering approaches require the insertion of short stretches of DNA that either encode for functional peptide tags^[55]^ – such as affinity tags for purification or detection,^[56]^ protease cleavage sites for on‐demand inactivation,[^57^, ^58^] tags for post‐translational modification via click chemistry,^[59]^ or labeling sites for protein visualization^[60]^ – or encode for molecular recognition sites required for further library processing; for example, restriction enzymes sites that can subsequently be used for creating systematic insertion or deletion libraries.

While tagging with functional peptides is usually done at the N‐ or C‐terminus of the protein, in certain instances this is impossible – in case the termini are functionally relevant or the functional insert needs to be inserted in the middle, as is the case for targeted inaction via protease cleavage^[58]^ – and proteins need to be screened for permissive sites that accept additional amino acids.

In the context of enzyme engineering, random insertion and deletion (indel) libraries have recently been shown to enhance the evolvability of proteins.^[61]^ Therefore, indel evolution could be a promising yet underexplored route towards new enzymatic function.

Indel libraries are traditionally created via transposon mutagenesis,[^61^, ^62^, ^63^] but those protocols are often time‐consuming and transposon insertion site bias^[64]^ compromises the creation of uniform and comprehensive indel libraries. Here, the above‐outlined array‐oligo pool‐based mutational protocols could become effective alternatives.

### 3.1 *In vitro* techniques for creating insertion or deletion libraries

PALS was used to create a comprehensive one amino acid deletion library of the yeast transcription factor Gal4.^[37]^ It is imaginable that PALS could also be used to systematically delete more than one residue or insert a short peptide tag, by encoding a tag or deletion in the middle of a mutagenic oligo. Similarly, it should also be feasible to create indel libraries using NM. An oligo‐encoded deletion or insertion approach (at one or a few sites) has shown feasible by the QuikChange™ protocol,^[65]^ but to the best of our knowledge, it has not yet been experimentally used to create comprehensive indel libraries using NM or PALS.

### 3.2 *In vivo* techniques for creating insertion or deletion libraries

PR was shown capable of generating a comprehensive insertion library (insertion of a given tag after each amino acid) for the entire open reading frame of the CRISPR protein Cas9 from *Streptococcus pyogenes* (SpCas9, 1368 amino acids).^[53]^ The insert in this case was a 6 bp restriction site encoding for either one of the two restriction sites NheI or SpeI (both libraries need to be created for their MISER method). Eventually, those sites were used to generate a comprehensive deletion library of SpCas9 with the goal to systematically size‐minimize this large protein to make it better suitable for medical and bioengineering applications; a general method that can systematically explore deletion‐landscapes of any given protein and which they call genetic **m**inimization by **i**terative **s**ize‐**e**xclusion and **r**ecombination (MISER).

To address the low mutagenic efficiency of PR (the frequency of plasmids containing an insert after one round of PR, versus the number of wild‐type plasmid), the authors use the restriction sites in the tag to sub‐clone an antibiotic resistance marker (Cm), allowing the experimenter to select for plasmids with an insert. In a second step this marker is cut out and the plasmid re‐ligated, yielding a 100 % mutagenized library. Of course, this strategy is only viable for tags encoding one or several restriction sites, but those could be either encoded or added to a given tag.

Further, CREATE was shown to enable the introduction of deletions (100 bp) in the genomically encoded *E. coli galK* gene with 70 % efficiency.^[42]^ Based on this efficiency and the overall scalability of CREATE,^[54]^ it is likely that customized (shorter or longer) (near)‐comprehensive deletion or insertion libraries can be created using this method.

## 4 Encoding Complex Libraries of Short Genes (up to 700 bp) via Oligo Pools

Once the number of required changes per open reading frame increases, site‐directed mutagenesis can become inefficient, and complete *de novo* synthesis of a gene is required. In enzyme engineering, this can be the case when a heterologous enzyme or a full pathway of multiple enzymes needs to be recoded for optimized codon usage or if various genes of a pathway need to be engineered together. One field that historically depended on the massive synthesis of new‐to‐nature DNA is the field of *de novo* protein design. Here, often >7000 computational designs need to be tested for sequence‐function relations. Not surprisingly, this field has early on developed techniques that use oligo pools for the synthesis of these designs.

In this section, we will introduce gene synthesis methods that are optimized to produce large libraries of different gene fragments in one pool. Those libraries are typically required during large‐scale functional testing of *de novo* designed proteins, and for targeted protein engineering at multiple sites. Those methods are less suitable for synthesizing long genes (>700 bp) or a pathway. Methods that are suitable to assemble a single gene or a multi‐gene pathway from oligo pools, rather than a library of variants, will be discussed in section 4.

### 4.1 Gene assembly in one pot without compartmentalization

In an effort to assemble 2271 designs of a short, 64 to 84 amino acid long protein domain, Klein *et al*. developed a method called multiplexed pairwise assembly (herein abbreviated as MPA) that allows assembling gene fragment libraries of ∼250 bp in length in joint‐pools of 250 to 2271 targets.^[38]^ Similar to the saturation‐mutagenesis methods reported above, MPA relies on relatively standard enzymatic molecular biology reactions for assembly. In pools of relatively low complexity (250 targets), MPA achieved the error‐free synthesis of on average 90.5 % of these 250 targets (% coverage, Table 3). Increasing the target pool complexity to the synthesis of the complete desired set of 2271 protein designs simultaneously in one‐pot, MPA still allowed the error‐free synthesis of 70.6 % of the 2271 targets (Table 3); and 11.8 to 31.3 % of all assembled molecules are error‐free (% accuracy, Table 3) Although not shown, one can imagine that the 250 bp fragments assembled via MPA can be assembled to longer genes using, for example, Golden Gate assembly.

**Table 3:** Performance overview of methods for gene library assembly (e. g. for testing various protein designs).

Method	Gene size (bp)	Pool size^[a]^	% coverage^[b]^	% accurate assemblies^[c]^	Ref.
MPA	192–252	131 to 250	72.7 to 96.4	11.8 to 31.3	[38]
	192–252	1212	84.2	11.8 to 31.3	[38]
	192–252	2271	70.6	11.8 to 31.3	[38]
DropSynth 2.0	675	384	92.0	23.5	[43]
	675	1536	80.0	22.6 to 27.6	[43]

[a] Number of independent assemblies performed in one pool. [b] Number of assemblies (per 100 designed assemblies) that show at least one error‐free molecule. [c] Number of error‐free assembled molecules per 100 assembled molecules.

MPA starts with designing the 250 bp target. Each target is then computationally divided into two partially overlapping fragments A and B (each fragment is 160 bp including the adaptors required for priming later in the protocol). Oligo encoded fragments are ordered containing one 3’ pool‐specific and one 5’ general adaptor for priming. This allows encoding all fragments in one oligo pool but to specifically amplify those that go into an assembly reaction together. The primers binding to the pool‐specific adaptors are uracil‐containing, such that these priming sites (as they would interfere with hybridization of A and B fragments) can be removed prior to assembly with Uracil Specific Excision Reagent (USER enzymes). The assembly is based on overlap extension PCR using the same outer primers as used for oligo amplification. Although deep sequencing revealed that between 70 % and 90.5 % (depending on the number of targets per pool) of the assembled targets had correct error‐free molecules represented in the pool of all molecules (Table 3), those error‐free molecules needed to be purified from the pool of all error‐containing molecules for downstream applications. Here the authors use dial‐out PCR, a method that allows identifying and amplifying error‐free assemblies to retrieve them for downstream applications.[^66^, ^67^] Specifically, in dial‐out PCR, single molecules get tagged with unique barcodes before sequencing. Error‐free molecules can then be identified via NGS and amplified via PCR using primers that bind to the unique barcodes of error‐free assemblies.

### 4.2 Compartmentalized assembly in water‐in‐oil droplets

A second method that can assemble thousands of genes from microarray‐derived oligos in a single reaction is DropSynth^[39]^ and its advanced version DropSynth 2.0.^[43]^ Here, fragments of ∼450–675 bp in length are assembled within water‐in‐oil droplets, allowing the experimenter to multiplex many assemblies. The concept is that all oligos required for one assembly get hybridized to a microbead using oligo‐barcodes. The oligo‐coated microbeads then get encapsulated in a water‐in‐oil droplet, creating spatially separate reactions.

DropSynth follows several steps: The genes to be synthesized are first bioinformatically split into several fragments, such that each fragment can fit onto one oligo. Restriction sites and a microbead barcode are also added to each oligo. Oligos are then amplified by PCR using a biotinylated primer, followed by digestion at high temperature to expose the microbead barcode as a single‐stranded DNA overhang. Processed oligos are mixed with a pool of either 384 or 1536 barcoded microbeads (limited by the number of unique barcodes), with each microbead containing only one complementary barcode sequence. Complementary oligos (all that are required for a specific assembly) hybridize and are ligated to the microbeads. The loaded beads are then mixed with PCR reagents, a restriction enzyme, and some fluorinated oil and vortexed to form a water‐in‐oil emulsion, which is placed into a thermocycler where the restriction enzyme displaces the oligos from the bead and the gene assembly reaction takes place inside the droplets. Upon completion, the aqueous solution containing the assembled genes is recovered from the emulsion and PCR‐amplified for downstream applications. The authors show that the optimized DropSynth 2.0 protocol^[43]^ can be used to build thousands of gene‐length fragments at >20 % accuracy, meaning that >20 % of the assemblies are error‐free, with a coverage of 80–92 % depending on the number of targets that were attempted to be assembled in one pool (% coverage, Table 3).

## 5 Synthesizing Individual Genes and Pathways

### 5.1 *In vitro*‐based methods for gene and pathway assembly

Over the last decade, several protocols have been developed that allow the assembly of genes and multi‐gene pathways from error‐prone array‐based oligo pools.[^18^, ^68^, ^69^, ^70^] They all involve three core steps: First, amplifying sub‐pools of oligos via pool‐specific primers; this is necessary to get enough oligos for the assembly. Second, assembly of sub‐pools by overlap PCR or ligase chain reaction, and third, an error removal step, that removes erroneous molecules. Error removal either involves sequencing‐based methods such as dial‐out PCR^[66]^ or it is based on depleting erroneous molecules via enzymatic cleavage or affinity‐based capture. The latter is based on the fact that during gene assembly a pool of perfect and imperfect double‐stranded sequences is produced. Melting and reannealing pairs perfect and imperfect strands and mis‐hybridized bases can be recognized by mismatch binding proteins or mismatch cleaving proteins. The efficiency of various enzymes and binding proteins has been systematically compared.^[71]^

Here we will highlight one gene‐ and pathway‐ assembly protocol that builds on available methods for each of the three above outlined steps, but is optimized to achieve a very low error rate (0.53 per kb) and most importantly can be implemented with regular molecular biology expertise and equipment:^[40]^ The authors use the full *de novo* synthesis of the 10‐gene lycopene biosynthetic pathway (11.9 kb) from 479 oligos (64 to 124 bp) as an example. The protocol uses PCR to amplify the entire oligo pool followed by a first error removal step via multiple consecutive annealing and MutS‐immobilized column purifications (so‐called MutS‐immobilized cellulose column, MICC).^[72]^ Error‐depleted oligos are then assembled into 500 bp fragments using ligase chain reaction. Residual errors are removed by a second round of MICC. The 500 bp parts are then assembled into the full pathway (encoded as three operons) using Gibson assembly.

### 5.2 Potential *in vivo* approaches for gene and pathway assembly

Besides the above outlined enzymatic gene assembly, it is well known that simple yeast assembly – based on *S. cerevisiae's* intrinsic capacity to perform homology‐repair‐based assembly of overlapping fragments ‐ can be used to assemble genes of >1000 bp length from short overlapping oligonucleotides.^[73]^ While in the original method the authors use column‐synthesized oligonucleotides at a scale of >10 nmol, it can be imagined that this method is viable using amplified next‐generation oligo pools, although this has not been shown yet.

## 6 Library Analysis by Second‐ and Third‐Generation Sequencing

Next‐generation protein engineering relies on the creation of large libraries often created in one pot and often created by methods that are not error‐free. As such, the created libraries must be properly evaluated for coverage, target mutation frequency, and off‐target mutation frequency, to calculate a sufficient screening sample size to ensure library coverage. Evaluation has mostly been done by short‐read NGS (also called second‐generation sequencing), such as Illumina‐based sequencing, because of the high throughput, low cost, and wide accessibility of the method. One limitation of second‐generation sequencing is the inherent short read length (75 to 500 nucleotides for Illumina sequencing platform). Therefore, a mutation that is located outside of the read‐window would be invisible in a library analysis. Because of these limitations, DMS or *de novo* protein‐coding is usually performed on small genes or subsets of genes (tiling) (Figure 1B). Several second‐generation sequencing approaches designed for protein engineering have been reviewed elsewhere.^[6]^

Here we only want to highlight methods that overcome the resolution window in second‐generation sequencing, and recent methods that improve so‐called long‐read (third‐generation) sequencing approaches and make them suitable for library analysis in protein engineering.

### 6.1 Increasing the resolution window of short‐read (second‐generation) sequencing‐based library analysis methods

A method called “subassembly” is powerful to overcome the limited read length in NGS as full‐length sequences of mutagenized clones can be obtained from short NGS reads.^[44]^ In subassembly, each mutant clone in a complex library is individually coupled to a random molecular tag. Paired‐end reads are obtained with one fixed end reporting the tag sequence, and one shotgun end derived randomly from the insert. Shotgun reads are then grouped by tag to yield an accurate full‐length consensus haplotype that is longer than the constituent reads and can detect random sequencing errors.

### 6.2 Increasing accuracy and throughput of long‐read (third‐generation) sequencing for library analysis

Read lengths longer than 500 bp have been possible for a while using single‐molecule real‐time (SMRT) sequencing or single‐molecule Nanopore sequencing (offered by PacBio Oxford Nanopore respectively), but both technologies show reduced throughput and reduced accuracy when compared to short‐read sequencing methods,^[74]^ which so far made them less suitable for library analysis.^[6]^

To enhance the accuracy of SMRT‐based sequencing, Waltenspül *et al*. recently developed a computational error correction workflow that eventually allowed them to use SMRT‐based sequencing for the full‐length sequencing of a G‐protein coupled receptor library (gene length >1000 bp).^[45]^ Like this, information of mutational linkage was maintained due to the fact that each read covers the full protein gene.

In addition to the above‐outlined accuracy improvements, Schlecht *et al*.^[46]^ and Kanwar *et al*.^[47]^ developed methods to increase the throughput of SMRT‐based sequencing by up to 5‐fold. They use protein‐encoding libraries of up to 870 bp in length as an example. The throughput enhancement was achieved by concatenating individual library members – either using Gibson assembly or Golden gate assembly – into longer fragments of up to five library members, which are then sequenced together, taking full advantage of the long reads that can be achieved by SMRT‐based sequencing.

These advancements in accuracy and throughput will likely make SMRT‐based sequencing a valid method of choice for the validation of protein engineering libraries.

## 7 Conclusions and Outlook

Here we summarized currently available methods that use cheap but error‐prone array‐synthesized oligo pools as a source of synthetic DNA for protein engineering libraries. These methods allow the creation of targeted or comprehensive mutagenic libraries required for deep‐mutational scanning, directed evolution, or rational protein engineering. Further, they allow the assembly of *de novo* designed gene libraries of several thousands of genes of up to 700 bp in length as well as the assembly of defined longer genes and pathways (Figure 1A).

All methods only require standard molecular biology expertise and equipment to create libraries and relatively standard bioinformatics expertise to analyze the NGS data. As such, they provide affordable access to next‐generation protein engineering libraries for many laboratories.

Combining the herein outlined methods with newly developed methods for oligo pool purification,^[26]^ should further enhance the capacity of oligo pools for protein and pathway engineering by enhancing the sequence accuracy of assemblies, a major bottle‐neck for scale‐up.

In addition to library creation methods, we highlight how these libraries can be evaluated for quality and coverage using next‐generation sequencing and bioinformatics (Figure 1B); specifically, we point to newly developed long‐read (second‐generation) sequencing workflows that overcome accuracy and throughput limitations formerly associated with those second‐generation methods in the context of library quality control. Being able to sequence longer (gene length) fragments with high accuracy and throughput facilitates the quality control and sequence analysis of gene‐ and pathway length libraries without the need for tiling it into shorter fragments.

In summary, in‐house array‐synthesized oligo pool‐based protein library creation and analysis comes of age and can enable many exciting next‐generation protein engineering endeavors in many laboratories.

## Conflict of interest

The authors declare no conflict of interest.

## Biographical Information

*Bastiaan Kuiper received his BSc and MSc degree in Biomolecular Sciences from the University of Groningen in 2021 where he worked on his research project in the molecular genetics group. He conducted a research internship at the Fukushima Medical University in the department of cell science, Japan. His research interests include structural biology, protein‐protein interactions, and fluorescent spectroscopy*.

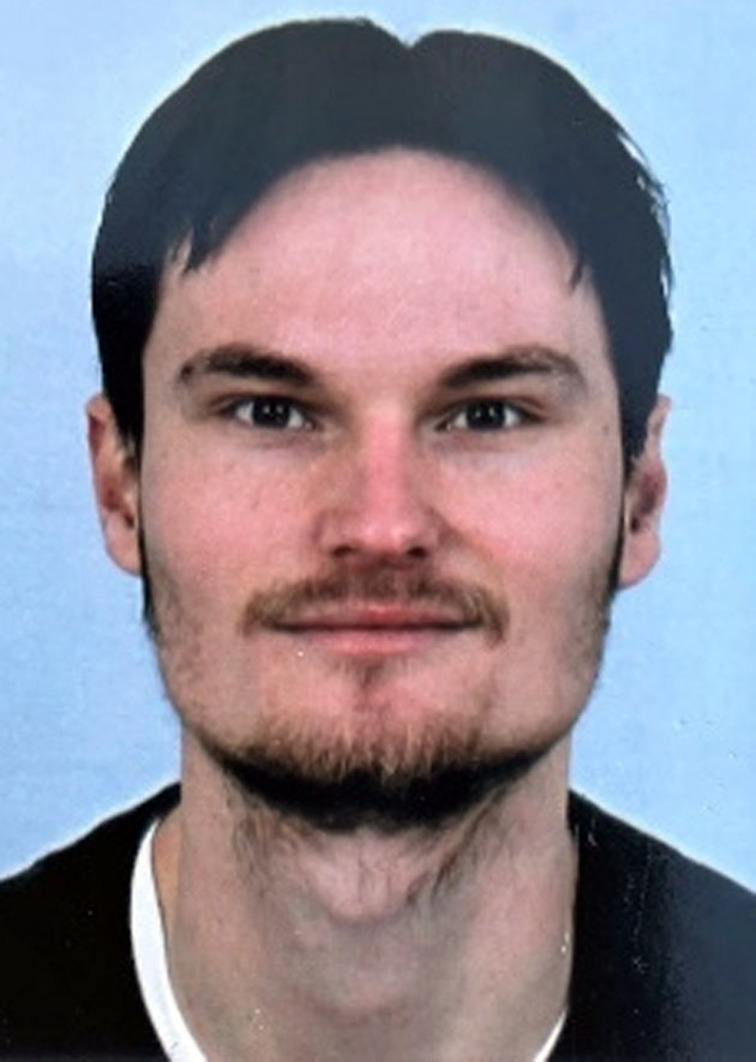

## Biographical Information

*Rianne C. Prins obtained her MSc in molecular cell biology from the University of Groningen in 2018 and is currently working on her PhD project at the Molecular Microbiology group at the Groningen Biomolecular Sciences and Biotechnology Institute, Faculty of Science and Engineering in Groningen, the Netherlands. Her PhD research is focused on sequence‐function relationships and protein engineering of yeast killer toxins*.

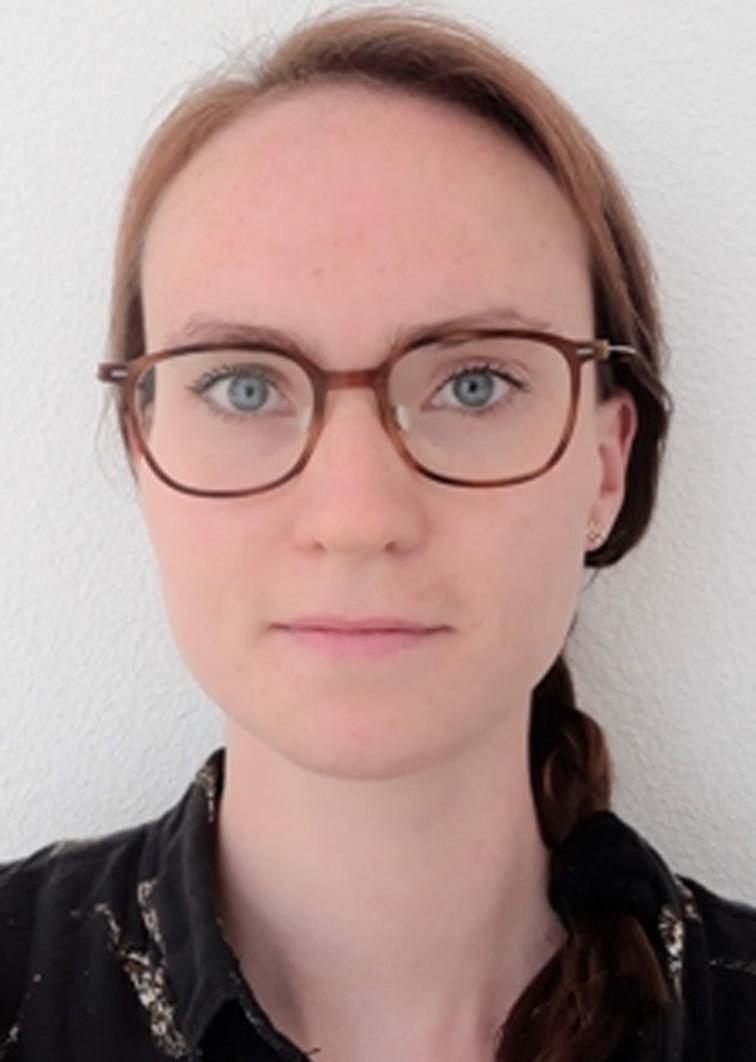

## Biographical Information

*Sonja Billerbeck holds a Master in Microbiology and Biochemistry from the University of Tübingen and the Max Planck Institute for Developmental Biology, and a PhD in Bioengineering from ETH Zürich. After postdoctoral work in yeast Synthetic Biology at Columbia University in New York City, she joined the University of Groningen as an Assistant Professor in 2019. Her laboratory uses a combination of synthetic biology, genome engineering, protein engineering, and environmental microbiology to access, understand and engineer the functional diversity of nature's yeast‐based mycobiome for applications in human health, industrial biotechnology and to answer fundamental questions on yeast (pathogen) biology*.